# Genome-Wide Investigation and Expression Profiling of HD-Zip Transcription Factors in Foxtail Millet (*Setaria italica* L.)

**DOI:** 10.1155/2018/8457614

**Published:** 2018-05-15

**Authors:** Wenbo Chai, Weina Si, Wei Ji, Qianqian Qin, Manli Zhao, Haiyang Jiang

**Affiliations:** National Engineering Laboratory of Crop Stress Resistance Breeding, Anhui Agricultural University, Hefei 230036, China

## Abstract

HD-Zip proteins represent the major transcription factors in higher plants, playing essential roles in plant development and stress responses. Foxtail millet is a crop to investigate the systems biology of millet and biofuel grasses and the HD-Zip gene family has not been studied in foxtail millet. For further investigation of the expression profile of the HD-Zip gene family in foxtail millet, a comprehensive genome-wide expression analysis was conducted in this study. We found 47 protein-encoding genes in foxtail millet using BLAST search tools; the putative proteins were classified into four subfamilies, namely, subfamilies I, II, III, and IV. Gene structure and motif analysis indicate that the genes in one subfamily were conserved. Promotor analysis showed that HD-Zip gene was involved in abiotic stress. Duplication analysis revealed that 8 (~17%) hdz genes were tandemly duplicated and 28 (58%) were segmentally duplicated; purifying duplication plays important roles in gene expansion. Microsynteny analysis revealed the maximum relationship in foxtail millet-sorghum and foxtail millet-rice. Expression profiling upon the abiotic stresses of drought and high salinity and the biotic stress of ABA revealed that some genes regulated responses to drought and salinity stresses via an ABA-dependent process, especially* sihdz29* and* sihdz45.* Our study provides new insight into evolutionary and functional analyses of HD-Zip genes involved in environmental stress responses in foxtail millet.

## 1. Introduction

The homeodomain leucine zipper (HD-Zip) gene family is a major group of transcription factors in the higher plants, which include a DNA-binding domain (HD) at the C-terminal and a leucine-zipper domain (bZIP also known as LZ domain) that can interact with other proteins [1, 2]. Through the leucine-zipper domain, HD-Zip proteins fold into homo- or heterodimers, which is necessary for DNA-binding specificity [3, 4]. Moreover, plants with an HD-Zip transgene exhibited improved morphological features when subjected to different stressful environmental conditions. The HD-Zip gene family can be divided into four classes (HD-Zip I–IV) according to their sequence similarity and structures of their different domains [5, 6]. A structural comparison found that all four classes contained the conserved HD and LZ domains, which promote dimerization, a prerequisite for DNA binding [6]. A minor distinction among the groups was also found; namely, HD-Zip I and HD-Zip II can recognize the pseudopalindromic DNA sequence CAAT(A/T)ATTG [3, 7], whereas HD-Zip III and HD-Zip IV recognize GTAAT(G/C)ATTAC and TAAATG(C/T)A, respectively [8, 9]. Furthermore, HD-Zip II contains two additional sets of five conserved amino acids, Cys, Pro, Ser, Cys, and Glu, known as CPSCG, and N-terminal consensus sequences. HD-Zip III and HD-Zip IV share a highly conserved lipid/sterol binding region called the START domain, followed by the start adjacent domain (SAD) [10, 11]. In addition, HD-Zip III proteins have the DNA-interacting PAS-related MEMKHLA domain at their C-terminus [12]. The expression level of HD-Zip IV is associated with the epidermis in general or specifically with its structure [13].

To date, HD-Zip-encoding genes have been analyzed in several plants, such as arabidopsis* (Arabidopsis thaliana)* [14], maize* (Zea mays)* [15], rice* (Oryza sativus)* [16], poplar* (Populus trichocarpa)* [17], cucumber* (Cucumis sativus)* [18], soybean* (Glycine max)* [19], and chrysanthemum* (Chrysanthemum morifolium)* [20]. Genes have different domains that enable them to participate in very diverse biological processes, but genes within the same family tend to have some functional conservation [6]. HD-Zip I proteins play important roles in plant responses to various environmental conditions, such as drought, exposure to abscisic acid (ABA), salinity stress, and extreme temperature. The* oshox22* gene belongs to HD-Zip I, the expression of which is strongly induced by salt stress, ABA, and polyethylene glycol (PEG) treatment, and weakly induced by cold stress [21]. HB1 in* Medicago truncatula* participates in responses to salt and osmotic stresses and is expressed in primary and lateral root meristem [22]. Zhao et al. also found that HD-Zip I genes were regulated by drought stress [15]. More recently, the maize HD-Zip I gene* Zmhdz10* was reported to positively regulate drought and salt tolerance in plants through an ABA-dependent signaling pathway [23]. In the case of HD-Zip II proteins, they are mainly involved in shade avoidance or light signaling, plant development, and abiotic stress tolerance [7]. For example, the expression of* ATHB2*,* HAT2*,* HAT3*, and* HAT4* is regulated by phytochrome, in that a low red/far-red light ratio rapidly induces the shade-avoidance response [24, 25]. Furthermore,* ATHB4* and* HAT3* play critical roles in establishing bilateral symmetry in cotyledons and developing leaves [25, 26].* HAHB10*, a sunflower HD-Zip II transcription factor, has also been shown to participate in the transition from the vegetative to the flowering stage and to mediate the content of phytohormones upon biotic stress [27].

HD-Zip III proteins were reported to act as regulators that participate in axial cell elongation, xylem differentiation, auxin transport, and lateral organ initiation [28, 29].* ZeHB13*, a HD-Zip III gene in* Zinnia*, is a pivotal transcriptional regulator responsible for early vascular development [30].* Arabidopsis* REV (REVOLUTA) can regulate meristem initiation at a lateral position [31]. In the case of HD-Zip IV proteins, these play crucial roles in epidermal development [32]; these proteins are thought to participate in trichome formation, lipid metabolism, and cuticle biosynthesis [33]. Maize OCL4 (outer cell layer 4) inhibits the patterning of the trichome and influences the division or differentiation of the anther [34].

Foxtail millet is a cultivated form of* Setaria*, the wild ancestor of which is green foxtail [35, 36].* Setaria* is an important food and biofuel in northern China [37, 38]. It is important to improve its product, which is associated with the environment. HD-Zip transcription factors can improve the morphological features upon exposure to stress. The key characteristics of* Setaria* are its small genome size, short life cycle, and close resemblance to maize and sorghum [39, 40]. High-quality sequencing of* Setaria* has been completed by Bennetzen et al. and Wang et al. [41, 42], which provided the foundation for genome-wide analysis of the HD-Zip genes. In this study, we found 47 protein-encoding genes in foxtail millet and 25 genes in green foxtail using BLAST search tools; we found that the putative proteins could be classified into four subfamilies. Structural and motif analyses were used to identify the characteristics of the HD-Zip genes. Then, Blast2GO, duplication, and microsynteny analyses were performed to predict the function of the genes in foxtail millet. To further identify the functions of the HD-Zip genes, expression profiling upon exposure to abiotic stresses such as drought and high salinity, and the biotic stress, ABA was performed. Overall, our results provide a foundation for further study of the functions of HD-Zip genes, particularly for members with potentially important functions in the responses to abiotic stresses, for application in molecular breeding.

## 2. Materials and Methods

### 2.1. Sequence Retrieval and Identification of HD-Zip Genes

The Hidden Markov Model (HMM) of* Homeobox-associated leucine zipper* (PF02183) was used to identify the foxtail millet HD-Zip genes in a protein database with the BLASTP program (*P* value = 0.001). Then, the obtained proteins were examined by Pfam (http://pfam.xfam.org/) [43] and SMART (http://smart.embl-heidelberg.de/) [44] for the presence of HD and Zip domains. The sequence IDs, protein sequences, genomic sequences, and CDS sequences were obtained from Phytozome (http://www.phytozome.net). Information about the physical parameters of the predicted HD-Zip proteins was obtained from the online ExPASy programs (http://web.expasy.org/protparam/), including the length of proteins, molecular weight (kDa), and isoelectric point.

### 2.2. Chromosomal Location, Gene Organization, and Conserved Motif Analyses

Chromosome information was obtained from Phytozome (http://www.phytozome.net). The chromosomal location map of the genes was generated by MapInspect (http://mapinspect.software.informer.com) and the genes were renamed according to their initial position on the chromosomes. To determine the gene structure, the intron-exon organization was analyzed by Gene Structure Display Server (GSDS; http://gsds.cbi.pku.edu.cn) via alignment of the CDS sequences with their corresponding genomic sequences. The online MEME program was used to perform the structure of motif (http://meme-suite.org/tools/meme) [45], using the default parameters. In addition, structure motif annotation was performed by Pfam and SMART.

### 2.3. Ka/Ks Values

Protein sequences of duplicated pairs were firstly aligned by MEGA v7.0 and then aligned sequences were used to calculate values of Ka/Ks by DnaSP v6.0. The Ks value was translated into duplication time in millions of years based on a rate of *λ* substitutions per synonymous site per year. The duplication time of duplicated genes was calculated by *T* = Ks/2*λ* × 10^−6^ Mya (*λ* = 6.5 × 10^−9^ for grasses) [10].

### 2.4. Phylogenetic Analysis, Gene Ontology (GO) Annotation, and Promoter Analysis

The protein sequences of HD-Zip were used to construct the phylogenetic tree with MEGA 7.0, using the neighbor-joining (NJ) method with 1000 bootstrap replications [46]. The GO annotation of the HD-Zip protein sequences was performed using Blast2GO (http://www.blast2go.com) [47], and the functions were defined into three categories: biological processes, cellular components, and molecular functions. The sequences about 2000 bp upstream of the transcription start site (ATG) were acquired using Phytozome. The online database PLACE (http://www.dna.affrc.go.jp/PLACE/signalscan.html) [48] was also employed to investigate putative* cis*-regulatory elements.

### 2.5. Modeling Construct of HD-Zip Proteins and Evolutionary Analysis of Paralogous and Orthologous Genes

The three-dimensional structures of HD-Zip proteins were used by Phyre2 server (Protein Homology/AnalogY Recognition Engine; http://www.sbg.bio.ic.ac.uk/phyre2) [49]. The position of the HD-Zip genes was marked by Perl script. The orthologous NAT genes among foxtail millet, maize, rice, sorghum,* Brachypodium distachyon*, green foxtail, and switchgrass were identified using OrthoMCL (http://orthomcl.org/orthomcl/) [50]. Then, the similarity of the orthologous genes was rechecked using local blast. The relationships of the orthologous pairs among the six species were plotted using Circos (http://circos.ca/) [51].

### 2.6. Expression Profiling of Tissues

To gain insight into the tissue-specific gene expression patterns of HD-Zip genes, the* Setaria italica* Illumina RNA-HiSeq reads for four tissues were retrieved from the European Nucleotide Archive [SRX128226 (spica), SRX128225 (stem), SRX128224 (leaf), and SRX128223 (root)]. Then, the low-quality reads were removed using the NGS toolkit (http://www.nipgr.res.in/ngsqctoolkit.html). The reads per kilobase per million (RPKM) values were used to normalize the mapped reads. A heat map was generated based on the RPKM values for each gene in all tissue samples using RNA-Seq data.

### 2.7. Plant Growth and Stress Treatment

The cultivar Yugu1 seedlings of foxtail millet were grown in a growth chamber under the following conditions: 28 ± 2°C with a 14-hour light/10-hour dark photoperiod. Different treatments were applied to 3-week-old plants. ABA stress was conducted by spreading 20 *μ*M ABA on the plants, and the plant leaves were harvested at 0, 1, 3, 6, 12, and 24 h after this treatment. For drought stress and salt stress, 25% PEG 6000 (dehydration) and 250 mM NaCl were used to water the plants, and the leaves were collected at 0, 1, 3, 6, 12, and 24 h. The samples were immediately frozen in liquid nitrogen and stored at −80°C until RNA extraction. Three biological replications were conducted per sample.

### 2.8. RNA Extraction and Quantitative Real-Time PCR Analysis

Total RNA was isolated following the method described by Huang et al. [52]. The quality and purity were assessed by 1.2% agarose gel and a NanoDrop ND-1000 spectrophotometer. The first-strand cDNAs were synthesized using RT-PCR Quick Master Mix (PCR-301; TOYOBO). The primers were designed by Primer3, and the NCBI database was used to identify their specificity. A constitutive Act2 gene-based primer was used as an endogenous control. The expression level was analyzed by the 2^−ΔΔCt^ method and the picture was drawn using SigmaPlot 10.0.

## 3. Results

### 3.1. Identification of the HD-Zip Genes in Foxtail Millet

High-quality sequencing has been completed in many species, which provides a foundation to study the HD-Zip gene family. The HMM sequence was used here to search for the foxtail millet HD-Zip genes in Phytozome. First, a total of 52 genes were found, after which all genes were confirmed by Pfam and SMART. By removing the different transcripts of the same gene, 47 nonredundant genes were identified in foxtail millet, which were named* sihdz1-sihdz47* based on their physical locations on chromosomes 1–9 (Table 1). The proteins have diverse lengths from 220 to 871 amino acids (aa), with an average of 506 aa. Moreover, the molecular weight ranged from 24.2 to 93.2 kDa and the isoelectric point varied from 4.6 to 9.63. All information on sihdz protein sequences is summarized in Table 1.

### 3.2. Phylogenetic Classification and Structure of HD-Zip in Foxtail Millet

**Figure 1:**
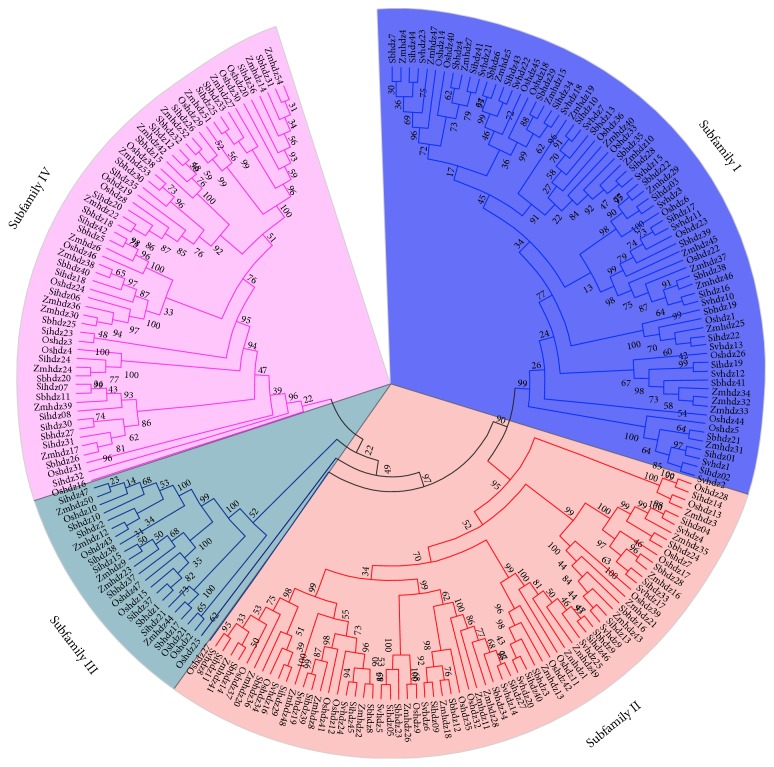
*Phylogenetic tree of HD-Zip transcription factors in foxtail millet, maize, rice, green foxtail, and sorghum*. The unrooted phylogenetic tree was produced with Clustal X2.0 by the neighbor-joining (NJ) method with 1000 bootstrap replications. The tree was divided into four subfamilies designated I to IV, with each HD-Zip family being indicated by a specific color.

To classify the HD-Zip genes in foxtail millet, the protein sequences in maize (54), rice (47), sorghum (41), and green foxtail (25) were all searched to generate an unrooted phylogenetic tree (Figure 1). Based on high bootstrap values, HD-Zip genes were divided into four subfamilies, I–IV. Subfamily III has the fewest HD-Zip genes, namely, only 22, while subfamily I has 68 genes, subfamily II has 67 genes, and subfamily IV has 57 genes.

**Figure 2:**
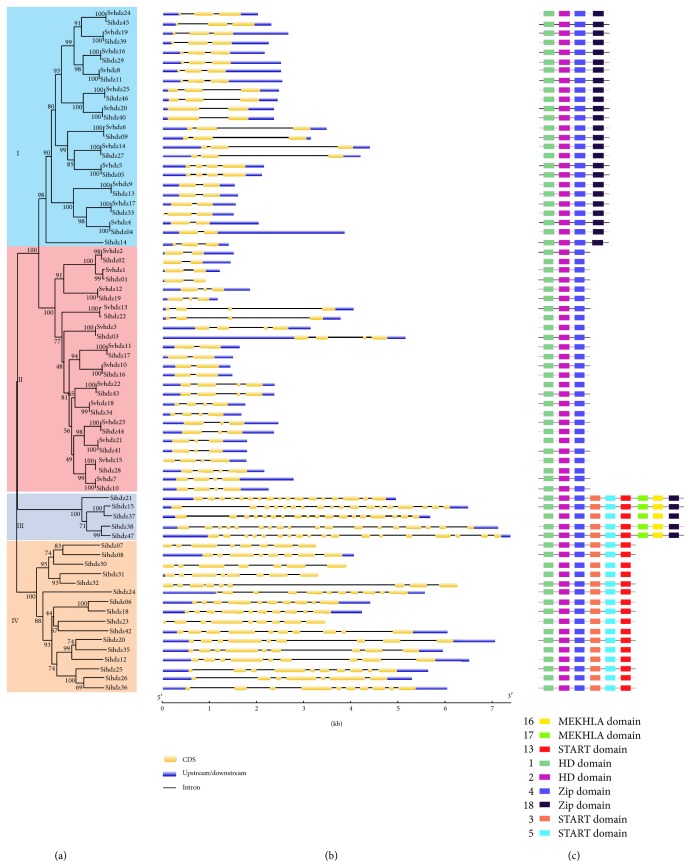
*Phylogenetic relationships, structure, and motif analysis of the 47 predicted foxtail millet and 25 green foxtail HD-Zip proteins*. (a) The tree was generated with the MEGA 7.0 program using the full-length amino acid sequence by the NJ method. Bootstrap values are indicated at the nodes. Each subfamily shown in the tree is marked with differently colored backgrounds. (b) Exon/intron structure of sihdz genes. Exons and introns are presented by green boxes and black lines, and untranslated regions (UTRs) are indicated by blue boxes. (c) Distribution of conserved motifs in foxtail millet HD-Zip family members. All motifs were identified using the MEME website. Differently colored boxes represent different motifs with their names on the right, and colored boxes are ordered manually according to the results of MEME analysis. The length of the box does not present the actual motif size in the protein.

To further investigate the evolutionary relationships among the 47 HD-Zip proteins in foxtail millet and 25 proteins in green foxtail, an unrooted phylogenetic tree was constructed by the NJ method, with 1000 bootstrap replications (Figure 2(a)). The HD-Zip genes were distinctly divided into the four subfamilies. In foxtail millet, subfamily IV had the highest number of members (16), followed by subfamily I and subfamily II, having the same number of members (13 each), while subfamily III had the lowest number of members. Interestingly, the green foxtail genes only clustered in subfamily I and subfamily II. Phylogenetic analysis revealed the presence of 18 gene pairs in foxtail millet, with strong bootstrap values (>91%).

The CDSs and their corresponding DNA sequences were used to investigate the structure of HD-Zip genes. The results indicate that the intron-exon structure conformed to the results of phylogenetic analysis (Figure 2(b)). Genes in each subfamily were shown to have very similar numbers of introns, such as subfamily I and subfamily II, each having from 1 to 3 introns, subfamily III having the most introns at 16-17, and subfamily IV having between 6 and 10.

The MEME online tool was used to identify the conserved motifs. A total of 25 motifs were investigated; the details of motif length and sequence are shown in Table 2. Every putative motif was annotated using Pfam and SMART. For simplicity, motifs that were not annotated are not shown in Figure 2. The results divided the HD-Zip gene family into four subfamilies, which were completely consistent with the classification from the phylogenetic analysis. Among these, motif 1 and motif 2 were found to encode the HD domain, and motif 4 and motif 18 were found to encode the LZ domain. The HD domain (motif 1 and motif 2) was found to be present in all 72 genes, while all of the genes had either motif 4 or motif 18. Subfamily III and subfamily IV include motif 3, motif 5, and motif 13 encoding the START domain. Motif 16 and motif 17, which encode the MEKHLA domain, were only found in subfamily III. In addition, it was suggested that some subfamily-specific motifs with no annotation may contribute to the diversification of the subfamily.

The Blast2GO program was used to search for the functions of HD-Zip proteins, including biological process, molecular role, and cellular component (Table S1). The results showed that sihdz proteins participate in diverse biological processes. HD-Zip I genes are usually involved in responses to abiotic stresses, ABA, and blue-light signaling. In contrast, HD-Zip II genes respond to auxins and illumination conditions. HD-Zip III and IV genes play essential roles in plant growth and development, such as meristem regulation, root development, and auxin transport. The molecular function analysis indicated that HD-Zip genes exert specific DNA-binding transcription activity. Furthermore, the cellular component analysis revealed that the products of HD-Zip genes are localized in the nucleus.

### 3.3. Chromosomal Location and Gene Duplication

**Figure 3:**
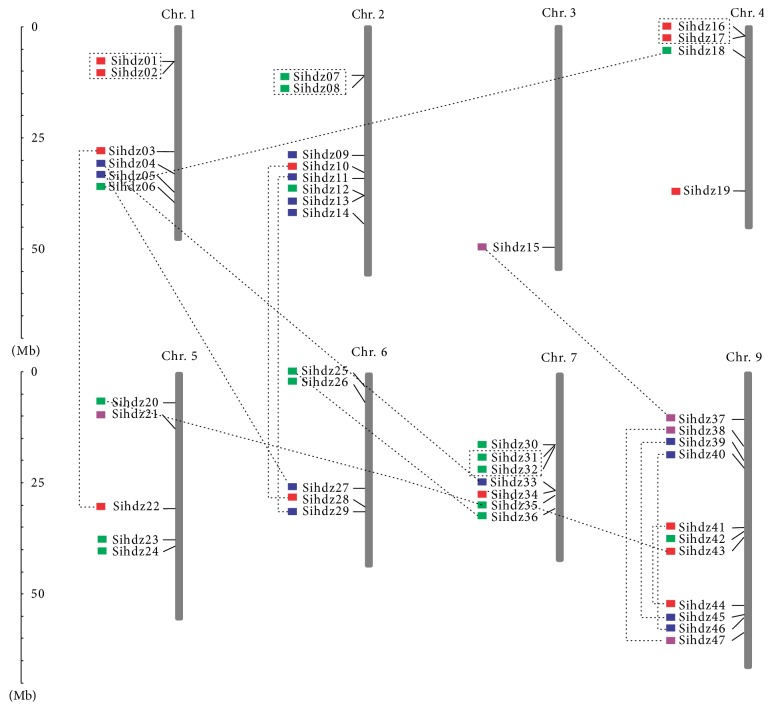
*Chromosomal distribution and segmental duplication events of 47 sihdz genes in nine foxtail millet chromosomes*. The 47 sihdz genes were mapped to the foxtail millet chromosomes. The genes in each subfamily are presented in different colors. The segmentally duplicated gene pairs are connected by lines.

The 47 HD-Zip genes are unevenly distributed along the nine foxtail millet chromosomes (Figure 3). The numbers of HD-Zip genes on each chromosome have a wide range. For example, chromosome 9 contains the highest number of genes (11), followed by chromosome 2 (8), while no genes are present on chromosome 8. Some chromosomes have regions of high gene density, such as chromosome 7 and chromosome 9. Tandem and segmental duplications are usually vital to gene expansion. A previous analysis of the whole genome revealed that gene duplications occurred approximately 70 million years ago [42]. Moreover, examination of gene duplication revealed that 4 gene pairs had been tandemly duplicated, whereas 14 gene pairs had been segmentally duplicated. The analysis was also performed in green foxtail; 2 gene pairs were found to be tandemly duplicated, while 6 gene pairs were found to be segmentally duplicated (Figure S1).

The ratio of nonsynonymous to synonymous mutation rates (Ka/Ks) was used to explore the selective constraints on duplicated sihdz genes. A value of Ka/Ks < 1 indicates that duplicated genes have undergone negative/purifying selection and Ka/Ks = 1 indicates that genes have been in a neutral state, while Ka/Ks > 1 signifies accelerated evolution with positive selection. The Ka/Ks values of the tandem duplications were <1, which means that these genes had been under purifying selection pressure. For a more thorough examination of paralogous pairs in maize, we constructed an NJ tree based only on foxtail millet proteins. Genes with more than 90% homology were considered to be paralogous pairs. A total of 18 gene pairs were identified. In this case, most of the duplicated genes had Ka/Ks < 1, meaning that purifying selection was vital to the functional divergence of sihdz genes. For* sihdz04/sihdz33*,* sihdz06/sihdz18*,* sihdz20/sihdz35*, and* sihdz34/sihdz43*, the Ka/Ks values were >1, so these genes may have played important roles in gene evolution (Table 3).

### 3.4. Promoter Analysis and Three-Dimensional Modeling of Sihdz

The* cis*-acting element of promoters is essential for determining tissue-specific expression and is involved in the regulation of gene expression under abiotic stress. An investigation of a region about 2000 bp upstream of the start codon (ATG) was used here to analyze* cis*-acting regulatory elements. The results showed that many stress-related elements participate in regulating gene expression. These include ABRE and motif IIb, which are involved in the response to abscisic acid; TCA element, which is involved in salicylic acid responsiveness; and MBS, which is an MYB binding site involved in drought inducibility. These elements were thought to play important roles in responses to abiotic stress, so these genes may be involved in responses to environmental conditions. The CAT box is a* cis*-acting regulatory element related to meristem expression. The CGTCA motif and the TGACG motif are involved in responses to MeJA stress. The GC motif is an enhancer-like element involved in anoxic-specific inducibility. The details of* cis*-acting elements are shown in Table S2, which provides a deeper understanding of the mechanism of stress tolerance in foxtail millet.

**Figure 4:**
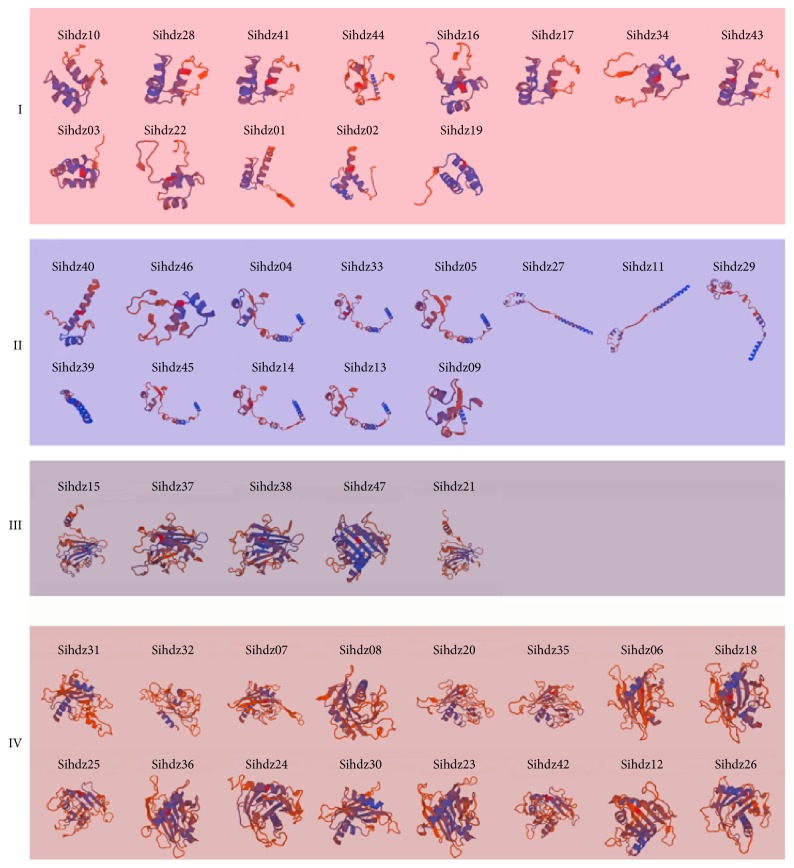
*Three-dimensional modeling of sihdz proteins*. The structure of sihdz proteins with a confidence level > 90% is shown and the activated sites are highlighted in blue.

Three-dimensional models of all sihdz proteins were constructed using the Phyre2 server (Figure 4). By comparing homologous gene structures, genes in the different subfamilies were shown to have different features. Subfamilies I and II have simple structures, while subfamilies III and IV have complex ones. Specifically, as well as *α*-helix and coil, subfamilies III and IV also have *β*-sheets. All of the predicted structures provide a foundation to analyze the functions of these molecules.

### 3.5. Orthologous Relationships of Sihdz Genes in Foxtail Millet, Maize, Rice, Sorghum, Brachypodium Distachyon, Green Foxtail, and Switchgrass

**Figure 5:**
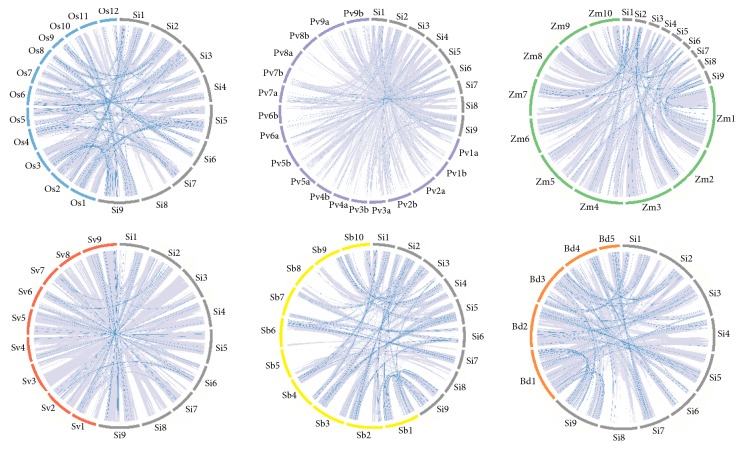
*Orthologous relationships among maize, rice, sorghum, Brachypodium distachyon, green foxtail, and switchgrass*. The numbers along the chromosome boxes indicate the sequence lengths in megabases. The grey line in the circle means the syntenic gene pairs in corresponding syntenic region. The blue lines represent syntenic relationships between the HD-Zip genes.

To further elucidate the relationships in foxtail millet, maize, rice, sorghum,* Brachypodium distachyon*, green foxtail, and switchgrass were chosen for further analysis; the orthologous genes were found using OrthoMCL (Figure 5). In total, 116 orthologous gene pairs were found in foxtail millet-rice, 114 were found in foxtail millet-sorghum, 74 in foxtail millet-maize, 61 in foxtail millet-green foxtail, 57 in switchgrass-millet, and 107 in* Brachypodium distachyon*-millet, which indicated that there were closer relationships among the monocotyledons. However, a closer relationship was found between foxtail millet and sorghum than between foxtail millet and maize, green foxtail, or switchgrass. These results are consistent with the findings of previous studies [35, 36, 38]. Some genes have no collinear genes, such as* sihdz02/GRMZM2G477415* and* sihdz02/Sobic.004G247500* being present in foxtail millet-maize and foxtail millet-sorghum, but* sihdz02* has no paralog in rice and* Brachypodium distachyon*, indicating that these orthologous pairs formed after rice and* Brachypodium distachyon* diverged from their common ancestor. Several genes have orthologs in all six species, so these genes may have been conserved over the course of evolution.

### 3.6. Expression Analysis of Foxtail Millet HD-Zip Genes in Various Tissues

To obtain insight into the expression patterns of sihdz genes in various tissues, a heat map was generated using the gene expression data (Figure S2). The tissue-specific expression data used here included those from root, leaf, stem, and spica. All of the 47 sihdz genes have various expression levels in different tissues.* Sihdz05* and* sihdz29* were highly expressed in all four tissues, while* sihdz02*,* sihdz24*,* sihdz26*,* sihdz34*,* sihdz01*,* sihdz51*,* sihdz48*,* sihdz40*,* sihdz33*,* sihdz32*,* sihdz28*,* sihdz25*,* sihdz22*,* sihdz20*,* sihdz07*, and* sihdz15* were rarely expressed in all tissues.* Sihdz21* showed high expression in spica and low expression in leaf, indicating that it has an important role in flowering.* Sihdz41* and* sihdz16* have high transcript accumulation in root, stem, and spica, which may be related to gene functions. Paralogous pairs with similar sequences exhibit divergent expression patterns. For example,* sihdz38* is highly expressed in spica, while* sihdz41* is highly expressed in root.

### 3.7. Expression Profiling of Sihdz Genes during Abiotic Stress

**Figure 6:**
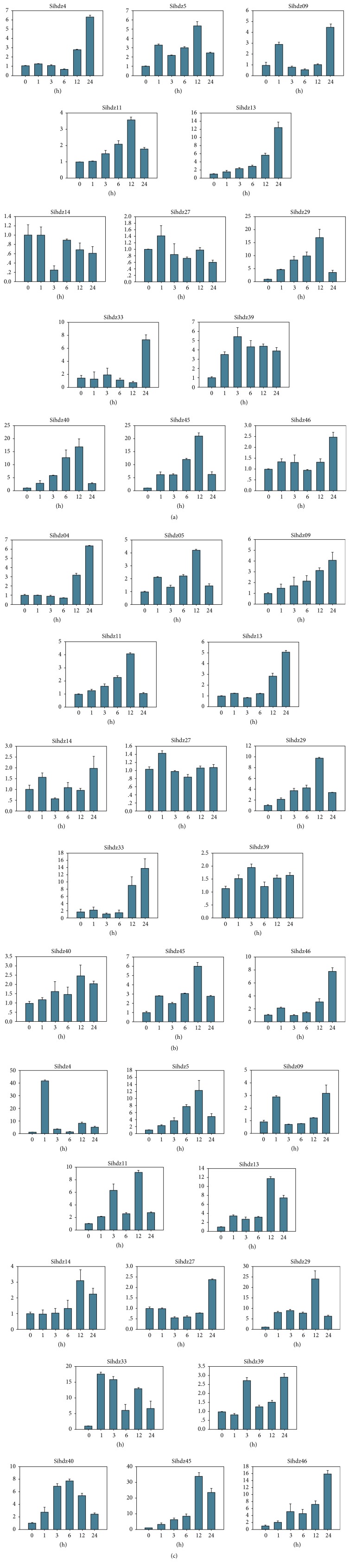
*Expression profiles of 13 selected HD-Zip I genes under drought, salinity, and ABA stresses using qRT-PCR*. (a) The expression profiles under PEG treatment. (b) The expression profiles under NaCl treatment. (c) The expression profiles under ABA treatment. The expression of each gene was calculated as a ratio relative to its expression in the control sample (0 h). Actin was used as an internal control to normalize the data.

For adaptation to several types of abiotic stress, such as dehydration, salinity, and ABA, the expression of many stress-related genes is induced. For example, previous studies reported that the expression of HD-Zip I genes was induced by drought [53, 54]. To gain further insight into the roles of HD-Zip genes upon exposure to drought and salinity, qRT-PCR was used to examine the expression profiles of these genes (Figure 6). The results showed that all of the HD-Zip I genes were drought- and salinity-responsive, with various expression patterns. Most of the HD-Zip I genes have the same expression profiles, such as* sihdz4*,* sihdz5*,* sihdz11*,* sihdz13*,* sihdz29*,* sihdz45*, and* sihdz46* (Figures 6(a) and 6(b)). During the early phase of salinity and drought treatment, the expression of* sihdz4* genes was steady, but then a drastic 7-fold change was observed at 24 h.* Sihdz29* showed a more than ~10-fold change in expression during the salinity and drought treatments at 12 h. In response to drought treatment, four genes showed the highest expression level, with a more than 12-fold change. We also compared the expression profiles of gene pairs; some of these pairs had similar expression profiles. For example, the expression of both* sihdz11* and* sihdz29* was upregulated nearly fourfold and peaked at 12 h and then was downregulated at 24 h.

The plant hormone abscisic acid (ABA) plays a vital role in drought and salt responses. During hormone treatment, all HD-Zip I genes were upregulated in ABA treatment (Figure 6(c)). Four genes exhibited minor changes in expression, with a relative expression scale from 0 to 5:* sihdz9*,* sihdz14*,* sihdz27*, and* sihdz39*. By contrast,* sihdz29* and* sihdz45* were clearly upregulated at all time points and peaked at 12 h (more than 25-fold change), while* sihdz09* and* sihdz46* peaked at 24 h (more than 15-fold change). Notably,* sihdz4* had the highest expression level at 1 h, approaching a 40-fold change. In summary, these genes may play important roles in biotic stress responses. The expression of* sihdz29* and* sihdz45 *was upregulated under the three treatments, so these two genes may play important roles in responses to environmental challenges.

## 4. Discussion

*Setaria* is a group of grasses that are important as biofuels. Many transcription factors have been performed, such as MYB transcription factor, AP2/ERF transcription factor, and the WD40 protein family [39, 55, 56]. These studies provided a foundation for evolutionary and functional characterization of transcription factors and new insight to improve the responses to environmental stimuli. The HD-Zip genes are a major group of transcription factors present in higher plants, which play significant roles in various biological processes during plant growth and development. The HD-Zip gene family has been researched systematically in maize and rice, but little is known about these genes in foxtail millet. We thus performed preliminary analyses of the HD-Zip gene family in foxtail millet, including its phylogeny and conserved motifs as well as three-dimensional modeling and* cis*-acting regulatory and expression profiling. In this study, a total of 47 nonredundant HD-Zip genes were found in the foxtail millet genome, while maize has 54, rice has 47, sorghum has 41, and green foxtail has 25 [12, 15, 57]. The numbers of HD-Zip genes were mostly equal among these grass species, with the exception of green foxtail; this means that the HD-Zip genes have been conserved over the course of evolution. Based on their phylogenetic tree, the putative genes were clustered into four distinct subfamilies. And the gene length in different subfamilies is conserved; the length of genes in subfamilies III and IV was longer than the genes in subfamilies I and II. The exon-intron analysis also indicated that the numbers of introns in paralogous genes were conserved. Some divergence in this regard was found in* sihdz40/sihdz46*,* sihdz05/sihdz27*, and* sihdz31/sihdz32*, indicating that a few insertions and deletions had accumulated. Subfamily I and subfamily II were found to have one to three introns, whereas subfamily III and subfamily IV had various numbers of introns, ranging from 6 to 17. Moreover, both the HD domain and the LZ domain were shown to be present in all HD-Zip genes. Subfamily III and subfamily IV contain a START domain, a highly conserved lipid/sterol binding region, while subfamily III also has a DNA-interacting PAS-related MEMKHLA domain at the C-terminus. Three-dimensional modeling analysis indicated that the genes in the same family tend to have similar protein structures. The structure and motif analysis results were consistent with the phylogenetic classification, which suggests that the classification of the genes is reliable.

**Figure 7:**
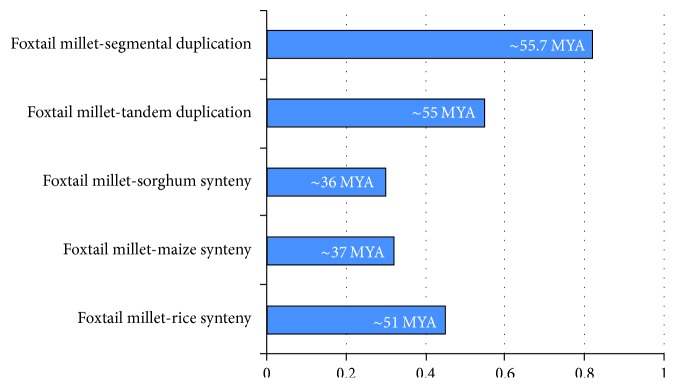
Time of duplication and divergence (MYA) based on synonymous substitution rate (Ks) estimated using orthologous and paralogous gene pairs between foxtail millet and sorghum, maize, or rice.

Duplications such as tandem and segmental duplication events have played dominant roles in the process of genome evolution. Such gene duplications have been reported in other gene families, such as WRKY and the CCCH-type zinc finger family [58, 59]. Our results obtained in this study indicate that segmental duplication has played important roles in HD-Zip gene expansion, especially in foxtail millet and green foxtail. The Ka/Ks values were used to measure the course in the gene duplication and divergence [60]. Analysis of paralogous pairs showed that the values are multiple from 0.067 to 2.4. And the homology modeling indicates that the paralog genes have similar structure; maybe paralog genes have similar function. The Ka/Ks ratios in four tandemly duplicated gene pairs ranged from 0.24 to 0.78, suggesting that negative or purifying selection is essential in HD-Zip gene duplication. Only four segmental gene pairs are under positive selection pressure, since their Ka/Ks ratios were estimated to be >1, which means that positive selection was active during the expansion of HD-Zip genes. We also estimated the Ka/Ks values of orthologous gene pairs in the genomes of four grasses. The average Ka/Ks value peaked between foxtail millet and rice (0.45), followed by foxtail millet and maize (0.32), and then foxtail millet and sorghum (0.30). This peak for foxtail millet and rice indicated that the HD-Zip genes diverged at an early stage, namely, around 51 MYA (Figure 7). The evolutionary analysis in foxtail millet between maize and sorghum suggests that intensive purifying selection has played a predominant role in the evolutionary process, as in other genes in foxtail millet [55]. The time of duplication in foxtail millet-maize is 37 MYA, while that in foxtail millet-sorghum is 36 MYA, which is in agreement with their Ka/Ks values (0.32, 0.30).

HD-Zip I genes have been demonstrated to play important roles in abiotic stress responses in rice and* Arabidopsis*, such as* Oshox22*, which was reported to participate in responses to ABA-mediated drought and salt tolerance [21]; moreover, overexpression of* ATHB5* in* Arabidopsis* indicated that it is a positive regulator of ABA responsiveness in seed germination and seedling growth [61]. However, no HD-Zip genes that respond to drought, salinity, and ABA have been reported in foxtail millet. As such, we investigated the expression patterns of millet HD-Zip I genes under conditions of drought, salinity, and ABA exposure. The results demonstrated that most of the genes were responsive to these three stresses. Specifically, in this study, the expression of* sihdz29* and* sihdz33* was strongly induced by drought, salinity, and ABA stresses, with increases of more than eightfold and tenfold. This may indicate that* sihdz29* and* sihdz33* regulate drought and salt tolerance through an ABA-dependent pathway. The gene pairs that have similar characteristics may have similar expression patterns, such as* sihdz4/sihdz33 *and* sihdz11/sihdz29*, regardless of drought or salt tolerance. Some divergence was also found, in that* sihdz40* drastically accumulated at 12 h (by more than 15-fold), while* sihdz46* exhibited minor expression changes under drought treatment. These findings of divergent expression patterns reveal that the HD-Zip genes may have experienced gene subfunctionalization over the course of evolution. Moreover,* sihdz4*,* sihdz5*,* sihdz11*,* sihdz13*,* sihdz29*,* sihdz40*,* sihdz45*, and* sihdz46* have similar expression patterns in response to drought and salinity, suggesting that these genes may be involved in defense against abiotic stress. Moreover, some genes exhibited minor expression changes when exposed to PEG treatment, such as* sihdz14*,* sihdz27*, and* sihdz46*, and when exposed to NaCl treatment, such as* sihdz14*,* sihdz27*,* sihdz39*, and* sihdz40*. We conclude that HD-Zip genes might play essential roles in regulating stress-responsive pathways for adaptation to the environment.

## 5. Conclusions

We identified 47 TFs in the foxtail millet genome. Phylogenetic reconstruction showed four subfamilies in the HD-Zip gene family: subfamilies I, II, III, and IV. Moreover, gene structure and motif analysis showed that genes within the same subfamily were conserved. Promotor analysis showed that HD-ZIP gene was involved in abiotic stress. Expression profile analysis by qRT-PCR revealed that* sihdz29* and* sihdz45 *may play important roles in regulating responses to multiple abiotic and biotic stresses such as ABA, NaCl, and PEG. These genes are thus potential targets for molecular breeding to improve plant tolerance. Our study also provided new insight into the evolutionary and functional characterization of HD-Zip genes in foxtail millet.

## Figures and Tables

**Table 1:** Feature list of 47 genes identified in foxtail millet and their sequence characteristics.

NIPGR ID	Phytozome Identifier	Physical position on foxtail millet genome			Protein length (aa)	PI	Molecular weight (Da)	Intron	Phylogeny group
		Chromosome	Start position (bp)	End position (bp)					
Sihdz01	Seita.1G087900	1	7798792	7799706	223	9.63	24184.04	1	I

Sihdz02	Seita.1G088000	1	7808171	7809619	220	9.58	23994.74	1	I

Sihdz03	Seita.1G201700	1	28159965	28165140	327	6.1	36098.44	3	I

Sihdz04	Seita.1G253700	1	32918259	32922133	236	5.4	26468.23	1	II

Sihdz05	Seita.1G306100	1	36983614	36985721	324	4.6	35439.06	3	II

Sihdz06	Seita.1G335800	1	39351189	39355603	711	6.2	77892.71	9	IV

Sihdz07	Seita.2G112400	2	10947386	10950661	735	6.4	79319.1	7	IV

Sihdz08	Seita.2G114000	2	11221021	11225092	712	6.21	78529.74	7	IV

Sihdz09	Seita.2G193200	2	28965749	28968902	303	4.65	32897.23	2	II

Sihdz10	Seita.2G224000	2	32601420	32603671	352	6.06	36851.92	3	I
	Seita.2G224000.2	2	32601420	32603671	419	9.69	43426.69		
	Seita.2G224000.3	2	32601420	32603671	313	9.32	33026.8		

Sihdz11	Seita.2G236800	2	34125151	34127695	265	4.82	28604.67	2	II

Sihdz12	Seita.2G282200	2	37895221	37901754	860	6.17	91157.98	8	IV

Sihdz13	Seita.2G284000	2	38029446	38031046	237	5.3	26082.92	1	II

Sihdz14	Seita.2G368600	2	44278030	44279425	230	6.02	25541.64	2	II

Sihdz15	Seita.3G395000	3	49640251	49646758	853	6.37	92896.04	17	III

Sihdz16	Seita.4G029000	4	1875175	1876650	271	9.4	28195.86	1	I

Sihdz17	Seita.4G029200	4	1896185	1897677	291	9.15	30526.21	1	I

Sihdz18	Seita.4G084600	4	6822689	6826925	699	5.89	75992.54	9	IV

Sihdz19	Seita.4G245400	4	36871689	36872853	223	9.28	24394.21	2	I

Sihdz20	Seita.5G079000	5	6743029	6750110	806	5.3	86426.43	8	IV

Sihdz21	Seita.5G141300	5	12454752	12459714	855	5.56	92530.12	16	III

Sihdz22	Seita.5G241600	5	30455176	30458970	224	9.35	24654.92	3	I

Sihdz23	Seita.5G326100	5	37528162	37531618	800	6.3	85890.61	8	IV

Sihdz24	Seita.5G344800	5	38943201	38948786	710	8.5	78461.92	10	IV

Sihdz25	Seita.6G039900	6	3018210	3023859	785	5.69	84606.48	9	IV

Sihdz26	Seita.6G072700	6	6484850	6490160	793	5.6	85138.95	6	IV

Sihdz27	Seita.6G145000	6	25873860	25878078	280	4.81	30601.69	2	II

Sihdz28	Seita.6G176400	6	30029766	30031922	355	7.06	36870.92	3	I
	Seita.6G176400.2	6	30029766	30031922	353	6.68	36614.61		

Sihdz29	Seita.6G187700	6	31175081	31177598	279	4.83	29580.76	3	II

Sihdz30	Seita.7G060900	7	16153965	16157882	715	6.07	78947.15	6	IV

Sihdz31	Seita.7G061100	7	16176368	16179683	730	6.01	80631.47	7	IV

Sihdz32	Seita.7G063300	7	16569209	16575494	674	6.3	74387.26	8	IV

Sihdz33	Seita.7G184600	7	26206329	26207830	335	6.6	37451.98	2	II

Sihdz34	Seita.7G187800	7	26435171	26436840	237	9.06	26043.72	3	I

Sihdz35	Seita.7G203300	7	27549926	27555890	793	5.87	85333.81	8	IV

Sihdz36	Seita.7G246700	7	30566314	30572371	781	5.48	84480.42	9	IV

Sihdz37	Seita.9G158800	9	10515531	10521227	856	6.16	93049.81	17	III

Sihdz38	Seita.9G219700	9	16264055	16271199	839	5.83	91968.72	17	III

Sihdz39	Seita.9G248400	9	19579512	19581764	299	5.1	32729.64	2	II

Sihdz40	Seita.9G258100	9	21176272	21178630	366	6.21	38925.15	1	II

Sihdz41	Seita.9G301000	9	34846306	34848095	247	8.69	27267.91	2	I

Sihdz42	Seita.9G309800	9	35775895	35781959	871	5.47	93247.34	9	IV
	Seita.9G309800.2	9	35775895	35781959	881	5.43	94315.57		

Sihdz43	Seita.9G321500	9	36997661	37000034	352	8.93	37940.91	3	I
	Seita.9G321500.2	9	36997661	37000034	347	8.79	37374.21		

Sihdz44	Seita.9G481800	9	52201230	52203593	278	8.07	29367.01	2	I

Sihdz45	Seita.9G511500	9	54329497	54331803	313	4.78	34357.78	2	II

Sihdz46	Seita.9G524200	9	55216547	55218989	335	6.07	36879.97	2	II

Sihdz47	Seita.9G572600	9	58368642	58376046	840	5.93	92167.29	17	III

**Table 2:** Predicted motif sequence in foxtail millet HD-Zip proteins.

Motif	Width	Best possible match
1	23	LGLEPRQVKFWFQNRRTRWKTKQ
2	29	KKKYHRHTKEQIQELERSFKECNHPDPKQ
3	35	YNGTIQLMYMEFYVPSPLVPTREFWFLRYCKQIED
4	15	LREENDRLKKEIDRL
5	29	CRRLPSGCLIQDMPNGYCKVTWVEHMEYD
6	29	MLILQESCTDASGSYVVYAPIDVNAMNVV
7	32	VFDYLRNEQRRGEWDIFSNGGQVQEMAHIANG
8	41	AVHFLYRPHFQSGQAFGARRWVASLQRQCEYMAILMSSNIP
9	45	GCLLTVAFQILVNSVPTAKLTLESVATVNSLICCTIEKIKAALKC
10	21	NGGDPDYVPLLPSGFAIIPDG
11	41	ENCQLRQENDKLRAENMTYKEAMRNPICPNCGGPAVLGEMS
12	25	GKRSMMKLAQRMMRSFCDAISGFNT
13	40	KPHGCHVEATRDCGVVIMTPTKLVEIFMDVNKWMEFFPCI
14	29	DKPMLLEIAERTMDEFMMMATKTEPLWVP
15	29	RKSVDDPGEPPGIVLSATTSVWLPVTPPH
16	50	MEQGYAYLPGGVCVSGMGRHVSYEQAVAWKVLGDDSNPHCLAFMFVNWSF
17	50	LKMFWHHSDAILCCSFKEKPMFTFANQAGLDMLETTLIALQDITLEKIFD
18	11	EVDCEYLKRCC
19	49	GGIICAKASMLLQNVPPAVLVRFLREHRSEWADYNIDAYSASALKANPC
20	41	SVPEVLRPLYESPKIVAQKMTTAALRHIRQIAHETSGEVPY

**Table 3:** Ka/Ks analysis and divergence between HD-Zip gene pairs in foxtail millet.

Gene	Duplicate	Ks	Ka	Ka/Ks	Purifying selection	Mya
Sihdz01	Sihdz02	0.2775	0.0662	0.238559	Yes	21.34615
Sihdz03	Sihdz22	1.7425	0.505	0.289813	Yes	134.0385
Sihdz04	Sihdz33	0.8217	1.9811	2.410977	No	63.20769
Sihdz05	Sihdz27	0.6551	0.3621	0.55274	Yes	50.39231
Sihdz06	Sihdz18	0.6382	1.0217	1.600909	No	49.09231
Sihdz07	Sihdz08	1.5288	1.3628	0.891418	Yes	117.6
Sihdz10	Sihdz28	0.4326	0.2743	0.634073	Yes	33.27692
Sihdz11	Sihdz29	0.4042	0.2187	0.541069	Yes	31.09231
Sihdz15	Sihdz37	0.6411	0.1003	0.15645	Yes	49.31538
Sihdz16	Sihdz17	0.7462	0.3158	0.423211	Yes	57.4
Sihdz20	Sihdz35	0.2327	0.2979	1.280189	No	17.9
Sihdz25	Sihdz36	0.8552	0.1837	0.214804	Yes	65.78462
Sihdz31	Sihdz32	0.3182	0.2121	0.666562	Yes	24.47692
Sihdz34	Sihdz43	0.6948	1.3737	1.977116	No	53.44615
Sihdz38	Sihdz47	1.0853	0.0731	0.067355	Yes	83.48462
Sihdz39	Sihdz45	0.561	0.466	0.83066	Yes	43.15385
Sihdz40	Sihdz46	0.46	0.2758	0.599565	Yes	35.38462
Sihdz41	Sihdz44	0.9095	0.2868	0.315338	Yes	69.96154

## Supplementary Materials

Supplementary Materials Table S1: functions of HD-Zip proteins searched by Blast2GO. Table S2:* cis*-acting element of promoter. Figure S1: chromosomal distribution and segmental duplication events of 25 sihdz genes in green foxtail. Figure S2: expression profiles of sihdz genes in different tissues. The RNA-Seq data were analyzed and a heat map was generated. Values from 0 to 7 represent low to high expression.
