# Supplementary material for: Genome-Wide Investigation and Expression Profiling of HD-Zip Transcription Factors in Foxtail Millet (Setaria italica L.)
Source: Biomed Res Int. 2018 May 15;2018:8457614. doi: 10.1155/2018/8457614 (PMC5976958; doi:10.1155/2018/8457614)
Supplement: Supplementary Materials — Table S1: functions of HD-Zip proteins searched by Blast2GO. Table S2: cis-acting element of promoter. Figure S1: chromosomal distribution and segmental duplication events of 25 sihdz genes in green foxtail. Figure S2: expression profiles of sihdz genes in different tissues. The RNA-Seq data were analyzed and a heat map was generated. Values from 0 to 7 represent low to high expression. [file 8457614.f1.zip › 8457614.f1/Supplementary Materials_BMRI_2222879.docx]

**Supplementary Material**

**Table S1. Functions of HD-Zip proteins searched by Blast2GO.**

| NIPGR ID | GO BIOLOGICAL PROCESS | GO MOLECULAR FUNCTION | GO CELLULAR COMPONENT | CLASSIFY |
| --- | --- | --- | --- | --- |
| Sihdz01 | regulation of transcription, DNA-dependent | DNA binding; transcription factor activity ; sequence-specific DNA binding | nucleus | Ⅰ |
| Sihdz02 | regulation of transcription, DNA-dependent | DNA binding; transcription factor activity ; sequence-specific DNA binding | nucleus | Ⅰ |
| Sihdz03 | regulation of transcription, DNA-dependent | DNA binding; transcription factor activity ; sequence-specific DNA binding | nucleus | Ⅰ |
| Sihdz10 | regulation of transcription, DNA-dependent | DNA binding | nucleus | Ⅰ |
| Sihdz16 | regulation of transcription, DNA-dependent | DNA binding; transcription factor activity ; sequence-specific DNA binding | nucleus | Ⅰ |
| Sihdz17 | regulation of transcription, DNA-dependent | DNA binding; transcription factor activity ; sequence-specific DNA binding | nucleus | Ⅰ |
| Sihdz19 | regulation of transcription, DNA-dependent | DNA binding; transcription factor activity ; sequence-specific DNA binding | nucleus | Ⅰ |
| Sihdz22 | regulation of transcription, DNA-dependent | DNA binding; transcription factor activity ; sequence-specific DNA binding | nucleus | Ⅰ |
| Sihdz28 | regulation of transcription, DNA-dependent | DNA binding; transcription factor activity ; sequence-specific DNA binding | nucleus | Ⅰ |
| Sihdz34 | regulation of transcription, DNA-dependent | DNA binding; transcription factor activity ; sequence-specific DNA binding | nucleus | Ⅰ |
| Sihdz41 | regulation of transcription, DNA-dependent | DNA binding; transcription factor activity ; sequence-specific DNA binding | nucleus | Ⅰ |
| Sihdz43 | regulation of transcription, DNA-dependent | DNA binding; transcription factor activity ; sequence-specific DNA binding; transcription activator activity | nucleus | Ⅰ |
| Sihdz44 | regulation of transcription, DNA-dependent | DNA binding; transcription factor activity ; sequence-specific DNA binding | nucleus | Ⅰ |
| Sihdz04 | regulation of transcription, DNA-dependent | DNA binding; transcription factor activity ; sequence-specific DNA binding | nucleus | Ⅱ |
| Sihdz05 | regulation of transcription, DNA-dependent | DNA binding; transcription factor activity ; sequence-specific DNA binding | nucleus | Ⅱ |
| Sihdz09 | regulation of transcription, DNA-dependent | DNA binding; transcription factor activity ; sequence-specific DNA binding | nucleus | Ⅱ |
| Sihdz11 | regulation of transcription, DNA-dependent | DNA binding; transcription factor activity ; sequence-specific DNA binding | nucleus | Ⅱ |
| Sihdz13 | regulation of transcription, DNA-dependent | DNA binding; transcription factor activity ; sequence-specific DNA binding | nucleus | Ⅱ |
| Sihdz14 | regulation of transcription, DNA-dependent | transcription factor activity; sequence-specific DNA binding |  | Ⅱ |
| Sihdz27 | regulation of transcription, DNA-dependent | DNA binding; transcription factor activity ; sequence-specific DNA binding | nucleus | Ⅱ |
| Sihdz29 | regulation of transcription, DNA-dependent | DNA binding | nucleus | Ⅱ |
| Sihdz33 | regulation of transcription, DNA-dependent | DNA binding; transcription factor activity ; sequence-specific DNA binding | nucleus | Ⅱ |
| Sihdz39 | regulation of transcription, DNA-dependent | DNA binding; transcription factor activity ; sequence-specific DNA binding | nucleus | Ⅱ |
| Sihdz40 | regulation of transcription, DNA-dependent | DNA binding; transcription factor activity ; sequence-specific DNA binding | nucleus | Ⅱ |
| Sihdz45 | regulation of transcription, DNA-dependent | DNA binding; transcription factor activity ; sequence-specific DNA binding | nucleus | Ⅱ |
| Sihdz46 | regulation of transcription, DNA-dependent | DNA binding; transcription factor activity ; sequence-specific DNA binding | nucleus | Ⅱ |
| Sihdz15 | regulation of transcription, DNA-dependent | transcription factor activity; sequence-specific DNA binding |  | Ⅲ |
| Sihdz21 | regulation of transcription, DNA-dependent | transcription factor activity; sequence-specific DNA binding |  | Ⅲ |
| Sihdz37 | regulation of transcription, DNA-dependent | transcription factor activity; sequence-specific DNA binding |  | Ⅲ |
| Sihdz38 | regulation of transcription, DNA-dependent | transcription factor activity; sequence-specific DNA binding |  | Ⅲ |
| Sihdz47 | regulation of transcription, DNA-dependent | transcription factor activity; sequence-specific DNA binding |  | Ⅲ |
| Sihdz06 | regulation of transcription, DNA-dependent | transcription factor activity; sequence-specific DNA binding |  | Ⅳ |
| Sihdz07 | regulation of transcription, DNA-dependent | transcription factor activity; sequence-specific DNA binding |  | Ⅳ |
| Sihdz08 | regulation of transcription, DNA-dependent | transcription factor activity; sequence-specific DNA binding |  | Ⅳ |
| Sihdz12 | regulation of transcription, DNA-dependent | transcription factor activity; sequence-specific DNA binding |  | Ⅳ |
| Sihdz18 | regulation of transcription, DNA-dependent | transcription factor activity; sequence-specific DNA binding |  | Ⅳ |
| Sihdz20 | regulation of transcription, DNA-dependent | transcription factor activity; sequence-specific DNA binding |  | Ⅳ |
| Sihdz23 | regulation of transcription, DNA-dependent | transcription factor activity; sequence-specific DNA binding |  | Ⅳ |
| Sihdz24 | regulation of transcription, DNA-dependent | transcription factor activity; sequence-specific DNA binding |  | Ⅳ |
| Sihdz25 |  |  |  | Ⅳ |
| Sihdz26 | regulation of transcription, DNA-dependent | transcription factor activity; sequence-specific DNA binding |  | Ⅳ |
| Sihdz30 | regulation of transcription, DNA-dependent | transcription factor activity; sequence-specific DNA binding |  | Ⅳ |
| Sihdz31 | regulation of transcription, DNA-dependent | transcription factor activity; sequence-specific DNA binding |  | Ⅳ |
| Sihdz32 | regulation of transcription, DNA-dependent | transcription factor activity; sequence-specific DNA binding |  | Ⅳ |
| Sihdz35 | regulation of transcription, DNA-dependent | transcription factor activity; sequence-specific DNA binding |  | Ⅳ |
| Sihdz36 | regulation of transcription, DNA-dependent | transcription factor activity; sequence-specific DNA binding |  | Ⅳ |
| Sihdz42 | regulation of transcription, DNA-dependent | transcription factor activity; sequence-specific DNA binding |  | Ⅳ |

**Table S2.** **Cis-acting element of promoter.**

| Site Name | Number of genes | Function of the cis-elements | Cis-elements types |
| --- | --- | --- | --- |
| CGTCA-motif | 42 | cis-acting regulatory element involved in the MeJA-responsiveness | Hormone responsive |
| TGACG-motif | 42 | cis-acting regulatory element involved in the MeJA-responsiveness | Hormone responsive |
| MBS | 40 | MYB binding site involved in drought-inducibility | Stress responsive |
| ARE | 33 | cis-acting regulatory element essential for the anaerobic induction | Stress responsive |
| ABRE | 30 | cis-acting element involved in the abscisic acid responsiveness | Hormone responsive |
| GC-motif | 29 | enhancer-like element involved in anoxic specific inducibility | Stress responsive |
| motif IIb | 25 | abscisic acid responsive element | Hormone responsive |
| CAT-box | 20 | cis-acting regulatory element related to meristem expression | Meristem expression |
| TC-rich repeats | 19 | cis-acting element involved in defense and stress responsiveness | Stress responsive |
| TCA-element | 18 | cis-acting element involved in salicylic acid responsiveness | Hormone responsive |
| TCA-element | 18 | cis-acting element involved in salicylic acid responsiveness | Hormone responsive |
| TGA-element | 18 | auxin-responsive element | Hormone responsive |
| GARE-motif | 17 | gibberellin-responsive element | Hormone responsive |
| HSE | 9 | cis-acting element involved in heat stress responsiveness | Stress responsive |

**Figure S1. Chromosomal distribution and segmental duplication events of 25 sihdz genes in green foxtail.**

**Figure S2. Expression profiles of sihdz genes in different tissues.** The RNA-Seq data were analyzed and a heat map was generated. Values from 0 to 7 represent low to high expression.
