# Supplementary material for: Genome-Wide Investigation and Expression Profiling of HD-Zip Transcription Factors in Foxtail Millet (Setaria italica L.)
Source: Biomed Res Int. 2018 May 15;2018:8457614. doi: 10.1155/2018/8457614 (PMC5976958; doi:10.1155/2018/8457614)
Supplement: Supplementary Materials — Table S1: functions of HD-Zip proteins searched by Blast2GO. Table S2: cis-acting element of promoter. Figure S1: chromosomal distribution and segmental duplication events of 25 sihdz genes in green foxtail. Figure S2: expression profiles of sihdz genes in different tissues. The RNA-Seq data were analyzed and a heat map was generated. Values from 0 to 7 represent low to high expression. [file 8457614.f1.zip › 8457614.f1/Supplementary Materials_BMRI_2222880.pdf]

Supplementary Material

Table S1. Functions of HD-Zip proteins searched by Blast2GO.

NIPGR ID	GO BIOLOGICAL PROCESS	GO MOLECULAR FUNCTION	GO CELLULAR COMPONENT	CLASSIFY
Sihdz01	regulation of transcription, DNA-dependent	DNA binding; transcription factor activity ; sequence-specific DNA binding	nucleus	Ⅰ
Sihdz02	regulation of transcription, DNA-dependent	DNA binding; transcription factor activity ; sequence-specific DNA binding	nucleus	Ⅰ
Sihdz03	regulation of transcription, DNA-dependent	DNA binding; transcription factor activity ; sequence-specific DNA binding	nucleus	Ⅰ
Sihdz10	regulation of transcription, DNA-dependent	DNA binding	nucleus	Ⅰ
Sihdz16	regulation of transcription, DNA-dependent	DNA binding; transcription factor activity ; sequence-specific DNA binding	nucleus	Ⅰ
Sihdz17	regulation of transcription, DNA-dependent	DNA binding; transcription factor activity ; sequence-specific DNA binding	nucleus	Ⅰ
Sihdz19	regulation of transcription, DNA-dependent	DNA binding; transcription factor activity ; sequence-specific DNA binding	nucleus	Ⅰ
Sihdz22	regulation of transcription, DNA-dependent	DNA binding; transcription factor activity ; sequence-specific DNA binding	nucleus	Ⅰ
Sihdz28	regulation of transcription, DNA-dependent	DNA binding; transcription factor activity ; sequence-specific DNA binding	nucleus	Ⅰ
Sihdz34	regulation of transcription, DNA-dependent	DNA binding; transcription factor activity ; sequence-specific DNA binding	nucleus	Ⅰ
Sihdz41	regulation of transcription, DNA-dependent	DNA binding; transcription factor activity ; sequence-specific DNA binding	nucleus	Ⅰ
Sihdz43	regulation of transcription, DNA-dependent	DNA binding; transcription factor activity ; sequence-specific DNA binding; transcription activator activity	nucleus	Ⅰ
Sihdz44	regulation of transcription, DNA-dependent	DNA binding; transcription factor activity ; sequence-specific DNA binding	nucleus	Ⅰ
Sihdz04	regulation of transcription, DNA-dependent	DNA binding; transcription factor activity ; sequence-specific DNA binding	nucleus	Ⅱ
Sihdz05	regulation of transcription, DNA-dependent	DNA binding; transcription factor activity ; sequence-specific DNA binding	nucleus	Ⅱ
Sihdz09	regulation of transcription, DNA-dependent	DNA binding; transcription factor activity ; sequence-specific DNA binding	nucleus	Ⅱ
Sihdz11	regulation of transcription, DNA-dependent	DNA binding; transcription factor activity ; sequence-specific DNA binding	nucleus	Ⅱ
Sihdz13	regulation of transcription, DNA-dependent	DNA binding; transcription factor activity ; sequence-specific DNA binding	nucleus	Ⅱ
Sihdz14	regulation of transcription, DNA-dependent	transcription factor activity; sequence-specific DNA binding		Ⅱ
Sihdz27	regulation of transcription, DNA-dependent	DNA binding; transcription factor activity ; sequence-specific DNA binding	nucleus	Ⅱ
Sihdz29	regulation of transcription, DNA-dependent	DNA binding	nucleus	Ⅱ
Sihdz33	regulation of transcription, DNA-dependent	DNA binding; transcription factor activity ; sequence-specific DNA binding	nucleus	Ⅱ
Sihdz39	regulation of transcription, DNA-dependent	DNA binding; transcription factor activity ; sequence-specific DNA binding	nucleus	Ⅱ
Sihdz40	regulation of transcription, DNA-dependent	DNA binding; transcription factor activity ; sequence-specific DNA binding	nucleus	Ⅱ
Sihdz45	regulation of transcription, DNA-dependent	DNA binding; transcription factor activity ; sequence-specific DNA binding	nucleus	Ⅱ
Sihdz46	regulation of transcription, DNA-dependent	DNA binding; transcription factor activity ; sequence-specific DNA binding	nucleus	Ⅱ
Sihdz15	regulation of transcription, DNA-dependent	transcription factor activity; sequence-specific DNA binding		Ⅲ
Sihdz21	regulation of transcription, DNA-dependent	transcription factor activity; sequence-specific DNA binding		Ⅲ
Sihdz37	regulation of transcription, DNA-dependent	transcription factor activity; sequence-specific DNA binding		Ⅲ
Sihdz38	regulation of transcription, DNA-dependent	transcription factor activity; sequence-specific DNA binding		Ⅲ
Sihdz47	regulation of transcription, DNA-dependent	transcription factor activity; sequence-specific DNA binding		Ⅲ
Sihdz06	regulation of transcription, DNA-dependent	transcription factor activity; sequence-specific DNA binding		Ⅳ
Sihdz07	regulation of transcription, DNA-dependent	transcription factor activity; sequence-specific DNA binding		Ⅳ
Sihdz08	regulation of transcription, DNA-dependent	transcription factor activity; sequence-specific DNA binding		Ⅳ
Sihdz12	regulation of transcription, DNA-dependent	transcription factor activity; sequence-specific DNA binding		Ⅳ
Sihdz18	regulation of transcription, DNA-dependent	transcription factor activity; sequence-specific DNA binding		Ⅳ

Sihdz20	regulation of transcription, DNA-dependent	transcription factor activity; sequence-specific DNA binding		Ⅳ
Sihdz23	regulation of transcription, DNA-dependent	transcription factor activity; sequence-specific DNA binding		Ⅳ
Sihdz24	regulation of transcription, DNA-dependent	transcription factor activity; sequence-specific DNA binding		Ⅳ
Sihdz25				Ⅳ
Sihdz26	regulation of transcription, DNA-dependent	transcription factor activity; sequence-specific DNA binding		Ⅳ
Sihdz30	regulation of transcription, DNA-dependent	transcription factor activity; sequence-specific DNA binding		Ⅳ
Sihdz31	regulation of transcription, DNA-dependent	transcription factor activity; sequence-specific DNA binding		Ⅳ
Sihdz32	regulation of transcription, DNA-dependent	transcription factor activity; sequence-specific DNA binding		Ⅳ
Sihdz35	regulation of transcription, DNA-dependent	transcription factor activity; sequence-specific DNA binding		Ⅳ
Sihdz36	regulation of transcription, DNA-dependent	transcription factor activity; sequence-specific DNA binding		Ⅳ
Sihdz42	regulation of transcription, DNA-dependent	transcription factor activity; sequence-specific DNA binding		Ⅳ

Table S2. Cis-acting element of promoter.

Site Name	Number of genes	Function of the cis-elements	Cis-elements types
CGTCA-motif	42	cis-acting regulatory element involved in the MeJA-responsiveness	Hormone responsive
TGACG-motif	42	cis-acting regulatory element involved in the MeJA-responsiveness	Hormone responsive
MBS	40	MYB binding site involved in drought-inducibility	Stress responsive
ARE	33	cis-acting regulatory element essential for the anaerobic induction	Stress responsive
ABRE	30	cis-acting element involved in the abscisic acid responsiveness	Hormone responsive
GC-motif	29	enhancer-like element involved in anoxic specific inducibility	Stress responsive
motif IIb	25	abscisic acid responsive element	Hormone responsive
CAT-box	20	cis-acting regulatory element related to meristem expression	Meristem expression
TC-rich repeats	19	cis-acting element involved in defense and stress responsiveness	Stress responsive
TCA-element	18	cis-acting element involved in salicylic acid responsiveness	Hormone responsive
TCA-element	18	cis-acting element involved in salicylic acid responsiveness	Hormone responsive
TGA-element	18	auxin-responsive element	Hormone responsive
GARE-motif	17	gibberellin-responsive element	Hormone responsive
HSE	9	cis-acting element involved in heat stress responsiveness	Stress responsive

Figure S1. Chromosomal distribution and segmental duplication events of 25 sihdz genes in green foxtail.

Figure S2. Expression profiles of sihdz genes in different tissues. The RNA-Seq data were analyzed and a heat map was generated. Values from 0 to 7 represent low to high expression.
