# Supplementary figures and images for: Genome-Wide Investigation and Expression Profiling of HD-Zip Transcription Factors in Foxtail Millet (Setaria italica L.)
Source: Biomed Res Int. 2018 May 15;2018:8457614. doi: 10.1155/2018/8457614 (PMC5976958; doi:10.1155/2018/8457614)

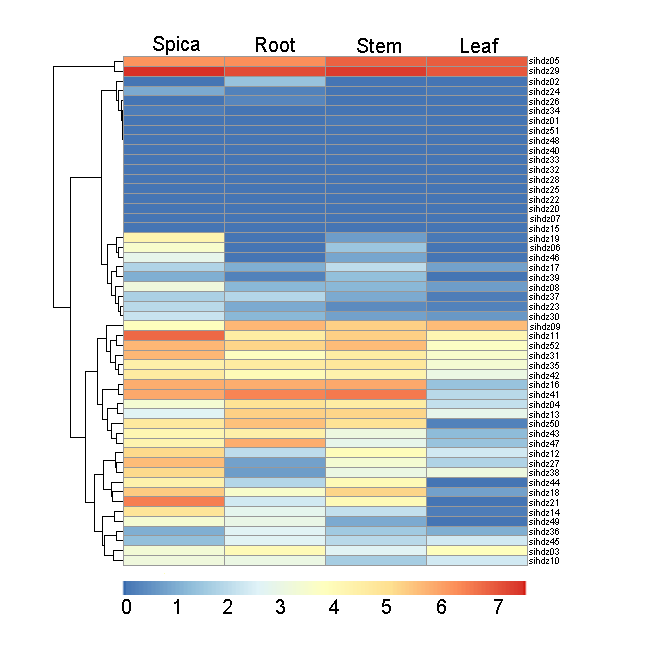

Supplement: Supplementary Materials — Table S1: functions of HD-Zip proteins searched by Blast2GO. Table S2: cis-acting element of promoter. Figure S1: chromosomal distribution and segmental duplication events of 25 sihdz genes in green foxtail. Figure S2: expression profiles of sihdz genes in different tissues. The RNA-Seq data were analyzed and a heat map was generated. Values from 0 to 7 represent low to high expression. [file 8457614.f1.zip › 8457614.f1/Figure S2_BMRI_2222892.tif]
